# Assessment of Drug Proarrhythmicity Using Artificial Neural Networks With *in silico* Deterministic Model Outputs

**DOI:** 10.3389/fphys.2021.761691

**Published:** 2021-12-10

**Authors:** Yedam Yoo, Aroli Marcellinus, Da Un Jeong, Ki-Suk Kim, Ki Moo Lim

**Affiliations:** ^1^Computational Medicine Laboratory, Department of IT convergence Engineering, Kumoh National Institute of Technology, Gumi, South Korea; ^2^R&D Center for Advanced Pharmaceuticals and Evaluation, Korea Institute of Toxicology, Daejeon, South Korea

**Keywords:** proarrhythmicity, toxicology classification, comprehensive *in vitro* proarrhythmic assay (CiPA), artificial neural network (ANN), *in silico*

## Abstract

As part of the Comprehensive *in vitro* Proarrhythmia Assay initiative, methodologies for predicting the occurrence of drug-induced torsade de pointes *via* computer simulations have been developed and verified recently. However, their predictive performance still requires improvement. Herein, we propose an artificial neural networks (ANN) model that uses nine multiple input features, considering the action potential morphology, calcium transient morphology, and charge features to further improve the performance of drug toxicity evaluation. The voltage clamp experimental data for 28 drugs were augmented to 2,000 data entries using an uncertainty quantification technique. By applying these data to the modified O’Hara Rudy *in silico* model, nine features (dVm/dt_max_, AP_resting_, APD90, APD50, Ca_resting_, CaD90, CaD50, qNet, and qInward) were calculated. These nine features were used as inputs to an ANN model to classify drug toxicity into high-risk, intermediate-risk, and low-risk groups. The model was trained with data from 12 drugs and tested using the data of the remaining 16 drugs. The proposed ANN model demonstrated an AUC of 0.92 in the high-risk group, 0.83 in the intermediate-risk group, and 0.98 in the low-risk group. This was higher than the classification performance of the method proposed in previous studies.

## Introduction

In 1999, the gastroprokinetic agent cisapride was recalled from the European pharmaceutical market because it was associated with torsades de pointes (TdP) (World health Organizations, 2001; Roden, 2008). In 2005, the International Council for Harmonization (ICH) established guidelines for the proarrhythmic assessment of drugs (Cavero and Crumb, 2005). This guideline suggests that the cardiotoxicity assessment for drugs should be conducted according to the S7B non-clinical evaluation and the E14 clinical evaluation guidelines. This conventional guideline requires extensive trials and has high sensitivity but low specificity for the risk classification of drugs. This means that even drugs that do not cause TdP are strictly regulated, negatively affecting drug development (Colatsky et al., 2016).

The Comprehensive *in vitro* Proarrhythmia Assay (CiPA) project was then established, with 13 advanced medical institutions attending the think-tank conference hosted by the FDA headquarters in 2013 to revise the existing drug development guidelines. The main change in the S7B non-clinical evaluation guideline through the CiPA project was the evaluation of the drug response of multiple ion channels using the *in silico* method from the hERG channel single analysis evaluation method through *in vitro* experiments (Crumb et al., 2016; Oh et al., 2018).

Dutta et al. (2017) proposed an *in silico* model that modified the human ventricular myocyte of Ohara’ Rudy (ORD) model (O’Hara et al., 2011). This optimized the maximum conductivities constant of the IKs, ICaL, IKr, INaL, and IK1 ion channels to 1.870, 1.007, 1.013, 2.661, and 1.698, respectively. This model corrected the maximum conductance of the underestimated or overestimated channels, allowing the drug response in the *in silico* model to be simulated similarly to that obtained *in vitro*. By deriving the qNet (the sum of the charge moving through six ion channels–INaL, ICaL, IKr, Ito, IK1, and IKs) using an *in silico* model, a feature for classifying the risk of TdP occurrence of drugs into high-, intermediate-, and low-risk levels was established (Dutta et al., 2017).

Parikh et al. (2017) performed simulations by applying drug effects to the ORD model to evaluate the drug response of multiple ion channels in a complex method (O’Hara et al., 2011). They extracted a group of TdP-inducing drugs from the derived results using a logistic regression technique. The 13 electrophysiological features (upstroke velocity, peak voltage, APD50, APD at −60 mV, APD90, resting voltage, AP triangulation, diastolic [Ca^2+^]i, the amplitude of CaT, peak [Ca^2+^]i, CaTD50, CaTD90, CaT triangulation) used as inputs to the logistic regression model were derived from *in silico* simulations under the condition of effective free therapeutic plasma concentration (EFTPC) and drug concentration when blocking the IKr channel by 50%. Classification scores under various basic cycle length (BCL) and ventricular tissue cell conditions (Epicardium, myocardium, Endocardium) were presented through a logistic regression-based classification model that classifies drugs that are TdP-induced or non-induced. In the EFTPC concentration condition, the classification performance was at least 76 points and up to 85 points, and in the hERG IC50 concentration condition, the performance was at least 77 points and up to 100 points (Parikh et al., 2017).

Li et al. (2019) classified the risk of drugs using qNet as an input to odds logistic regression model (Dutta et al., 2017). qNet was calculated using the model that added the hERG dynamic model to the modified ORD model (Dutta et al., 2017). The risk groups of drugs were classified into two qNet thresholds; the threshold for dividing the high-risk and intermediate-risk groups was 0.0579 μC/μF while the threshold for dividing the intermediate-risk and low-risk groups 0.0689 μC/μF. The classification accuracy was improved by incorporating the hERG-dynamic model into the analyses (Li et al., 2019). However, the disadvantages of this model include the large amount of data processing and mathematical complexity, such as *in vitro* experiments for parameter evaluation and quantification of uncertainty.

Llopis-Lorente et al. (2020) proposed a new method for classifying drug risk groups using nine decision trees. Three features were used as inputs. The first is T_*x*_ (Romero et al., 2018), which is the ratio of the drug concentration when action potential duration 90% (APD90)increased by 10% and the EFTPC concentration of each drug was calculated using the model proposed by Dutta et al. The second feature, T_qNet_, is the ratio of the calculated qNet value at 10 times the EFTPC concentration and the qNet value at steady state (Dutta et al., 2017; Llopis-Lorente et al., 2020). The third feature, T_*triang*_, is the ratio of the difference between APD90 and APD30 calculated at 10 times the concentration of EFTPC and calculated at steady state. The classification accuracy of the drug risk groups was 0.899 when T_*x*_ was used as an input, 0.908 when T_*triang*_ was used, and 0.917 for T_qNet_ (Llopis-Lorente et al., 2020).

In summary, previous studies typically derived a single feature such as APD90, qNet, or qInward which is considered to be highly correlated with TdP risk and used them as biomarkers to predict proarrhythmic drug by using binary classification methods. However, the traditional binary classification methods such as logistic regression and decision trees are simple linear classification algorithm and not suitable to classify categorical labels (Ismail et al., 2020).

Therefore, in this study, to increase the accuracy of drug toxicity assessments, we propose a drug TdP induced risk level classification model based on an artificial neural network (ANN). This model has nine multiple input features that all consider the action potential (AP) morphology, calcium transient morphology, and charge features.

## Methods

### Software and Data: Hill Fitting and Bootstrap

For this study, the same data fitting method and *in silico* model used by Li et al. (2019) were implemented based on the C++ language. We used the patch-clamp experiment data uploaded to the GitHub website^1^ from the CiPA project group (Crumb et al., 2016; Chang et al., 2017). Hill fitting was performed using the experiment data of the six ion channel patch clamps of the drug presented by Crumb et al. (2016). To quantify the uncertainty of the experimental data, we extracted 2,000 Hill coefficients and IC50 values for six ion channels by bootstrapping within 95% of the confidence interval using the Markov chain Monte Carlo (MCMC) model proposed by Chang et al. (2017). The MCMC model derives the optimal Hill curve using the least square method by inputting the experimental data such as drug concentration, ion channel block percentage, and pace. Based on the optimal Hill curve, 2,000 Hill curves within 95% of the confidence interval are derived (bootstrapped) to extract IC50 and Hill coefficients. Hill coefficients and IC50 values modified the conductivity of the six ion channels, and the resulting conductivity of these modified ion channels was applied to the *in silico* simulations. Information on the IC50 and Hill coefficients used can be found in the Supplementary Material.

### *In silico* Simulation Protocol

The ORD model, modified by Dutta et al. (2017), was used as an electrophysiological *in silico* model for cardiomyocytes (O’Hara et al., 2011). We selected 28 drugs to devise a classification model for the risk groups of drugs according to the CiPA project group (Li et al., 2019). These drugs consist of eight high-risk, eleven intermediate-risk, and nine low-risk groups, depending on the risk of drug-induced cardiac arrhythmias (Table 1). The ion conductance of the *in silico* model was modified through the inhibition factor (*^Equation^* ^1)^, and 2,000 samples of IC50 values and Hill coefficients obtained from each drug *via* Hill fitting and bootstrap were applied as inputs to the inhibition factor equation (Li et al., 2019). The concentrations of the drugs were set at 1, 2, 3, and 4 times the maximum serum concentration (free C_max_), which are the characteristic values of each drug. In total, 1,000 stimuli were applied with a stimulation period of 2 s, and stimuli duration of 0.1 ms for 2,000 possible drug-affected *in silico* models under four different concentrations of each drug.


(1) $$\text{I}\mathrm{nhibition}\mathrm{factor}=\text{[}\frac{1}{1+{\left(\frac{I\,C\,50}{\left[D\right]}\right)}^{h}}]{}^{-1}$$

Where IC50 is drug concentration for the 50% inhibition of ion current, D is the drug concentration, and h is the Hill coefficient.

**TABLE 1:** Twenty- eight drugs selected by the CiPA research group into high, intermediate, and low risk levels according to the possibility of causing Tdp (Li et al., 2019). Twelve drugs were used during the machine learning training and sixteen drugs were used during testing.

Used \risk level	High	Intermediate	Low
TRAINING	Quinidine	Cisapride	Verapamil
	Sotalol	Terfenadine	Ranolazine
	Dofetilide	Chlorpromazine	Diltiazem
	Bepridil	Ondansetrom	Mexiletine
TESTING	Disopyramide	Clarithromycin	Metoprolol
	Ibutilide	Clozapine	Nifedipine
	Vandetanib	Domperidone	Nitrendipine
	Azimilide	Droperidol	Tamoxifen
		Pimozide	Loratadine
		Risperidone	
		Astemizole	

### Feature Evaluation

Nine features related to TdP were derived through single-cell electrophysiology simulations. These features include AP features, calcium features, and ion charge features. Among the AP features, the AP duration 90 (APD90) is the duration between the depolarization point and the repolarization point 90% below the maximum amplitude in the curve of the AP. APD50 is the duration between the depolarization and repolarization points 50% below the maximum amplitude in the AP shape. dVm/dt_max_ is the maximum slope when the membrane potential is depolarized in the shape of the AP, and AP_resting_ is the resting membrane potential. Calcium features include calcium transient duration 90 (CaD90), which is the duration between 90% or less of the maximum amplitude during the transient period of influx calcium. CaD50 is the duration between 50% or less of the maximum amplitude during the influx calcium transient. Ca_resting_ is defined as the diastolic concentration of intracellular calcium. The qNet of the ion charge features is the total amount of ion charges that pass through the six ion channels (INaL, ICaL, IKr, IKs, IK1, Ito) until the end of the BCL, and is calculated as the sum of the integral of the current graph over time (Equation 1). qNet was described by Li et al. (2019) and was used as an input feature to classify the risk of TdP-induced drugs using a logistic regression model performed by Li et al., where qInward is the amount of charge change through the ICaL and INaL ion channels during the AP beat induced by the drug (Equation 3; Chang et al., 2017).


(2) $$\mathrm{qNet}=\int_{0}^{B\,C\,L}\left(I\,N\,a\,L+I\,C\,a\,L\,I\,K\,r+I\,K\,s+I\,k\,1+I\,t\,o\right)\,dt$$

(3) qInward=(ICaL_drug_AUC/ICaL_control_AUC+INaL_drug_AUC/INaL_control_AUC)/2

Where _drug_AUC is the area under the current change graph over time of each ion channel upon drug administration conditions and _control_AUC is the area under the current change graph over time of each ion channel in drug-free conditions. The criteria for selecting APs for feature calculation is that they should be the one AP with the highest dVm/dt_*max_repol*_ value during repolarization, except if depolarization or repolarization failed for the last 250 APs out of 1,000 APs (Chang et al., 2017). That is, one AP shape having the largest value is selected by comparing the slope value of the repolarization period of 250 AP shapes. Nine features were calculated from the selected AP. An *in silico* simulation was performed for each drug concentration (C_max_, C_max_*2, C_max_*3, C_max_*4), and the average value of the calculated nine features was assigned as the input of the ANN model.

### Artificial Neural Network Model

**FIGURE 1:**
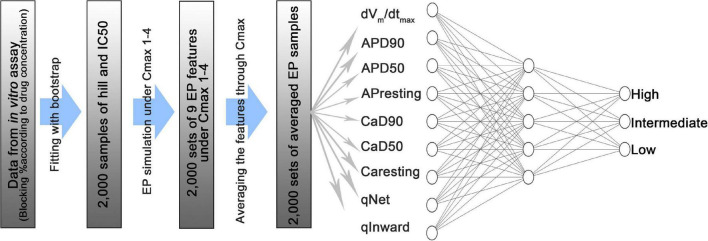
Schematic diagram of an artificial neural network model consisting of an input layer with 9 nodes, a hidden layer with 5 nodes, and an output layer with 3 nodes. dVm/dt_max_ is the maximum slope when the membrane potential is depolarized in the shape of the action potential; APD90 is the duration between the depolarization point and the repolarization point 90% below the maximum amplitude in the shape of the action potential; APD50 is the duration between the depolarization and repolarization points 50% below the maximum amplitude in action potential shape; AP_resting_ is the resting membrane potential; CaD90 is the duration between 90% or less of the maximum amplitude during the transient period of intracellular calcium; CaD50 is the duration between 50% or less of the maximum amplitude during the intracellular calcium transient; Ca_resting_ is the diastolic concentration of intracellular calcium; qNet is the total amount of ion charges that have moved through the six ion channels (INaL, ICaL, IKr, IKs, IK1, Ito) during the action potential duration; qInward is the average of the ratio between the drug reaction and the steady state of charges directed to the cell through the ICaL and INaL ion channels during the action potential period.

As proposed by CiPA, 12 drugs were used for model training, and 16 drugs were used for testing (Colatsky et al., 2016; Li et al., 2019; Figure 1). The proposed ANN model is composed of an input layer with nine nodes that considered nine features (dVm/dt_max_, AP_resting_, APD90, APD50, Ca_resting_, CaD90, CaD50, qNet, and qInward) as inputs, a hidden layer with five nodes, and an output layer with three nodes (Figure 1). The physical quantity and unit of the nine inputs are different. Their range is significantly different; While APD ranges from 360 to 800 ms, the qNet ranges from 0.03 to 0.08. nine input values were normalized using the “MinMaxscaler” function. “ReLu” was used as the activation function of the hidden layer, and the output layer is a categorical output layer having three nodes, and the probability at each node is calculated by the SoftMax activation function. We performed leave-one-out cross-validation to optimize the model during training. This is a validation method that excludes 12 training drugs one by one in order. One excluded drug was used as validation data and while the remaining 11 drug data were used as training data. Leave-one-out cross validation allows us to derive the most optimized model that is less affected by the extreme tendency of drugs to elicit the effect of interest. “Categorical_crossentropy” was used to calculate the difference between the true class and the predicted class when learning an ANN with an output layer having multiple labels. “Adam” is a method of calibrating weights and biases during backpropagation to minimize errors in the predicted class calculated through training. The learning rate was set at 0.01, the batch size was set to 32, and the Epoch was set to 100. The ANN code is available along with this publication at https://github.com/Yedam-Y/ANN_CiPA/. The data for ANN learning were acquired through *in silico* simulations. Since 2,000 samples were obtained for each drug and 12 drugs were obtained, a total of 24,000 samples were used. Data for the ANN test were obtained from the 16 drugs. There are 16 test drugs each with 2,000 samples. One dataset is made by randomly extracting one sample from each of the 16 drug groups, and 10,000 datasets were created in this study. With these 10,000 datasets, 10,000 ANN classifications were performed to obtain 10,000 AUC values. The visual data explaining this is shown in Supplementary Figure 2 (Li et al., 2019). As a test result, the performance of the model was evaluated using the area under the curve (AUC) corresponding to the area of the receiver operating characteristic (ROC curve). Here, 95% of the AUC and likelihood rate confidence intervals and the median of the frequency distribution of 10,000 AUC results were set as representative values to compare the results. Sensitivity and specificity are solved as in Equations 4, 5. Based on the sensitivity and specificity, the likelihood ratio is calculated (Equations 6, 7; Simundic, 2009). To prevent the denominator becoming 0, a value of 10^–3^ was subtracted from the result of the specificity.


(4) Sensitivity=True⁢positive/(True⁢positive+False⁢negative)

(5) Specificity=True⁢negative/(True⁢negative+False⁢positive)

(6) LR+=sensitivity/(1-specificity)

(7) LR-=(1-sensitivity)/specificity

For example, True positive means the number of drugs correctly classified among high-risk drugs. False positive refers to the number of drugs that are not in the high-risk group and are incorrectly classified as high-risk drugs. True negative refers to the number of drugs classified as non-high-risk drugs as non-high-risk. False negative refers to the number of drugs in a high-risk group that are incorrectly classified. Sensitivity is the ratio of classifiers to positive among the actual positives, and specificity is the ratio of classifiers to negative among the actual negatives. The positive likelihood rate is denoted as LR+ and the negative likelihood rate is denoted as LR−.

## Results

**FIGURE 2:**
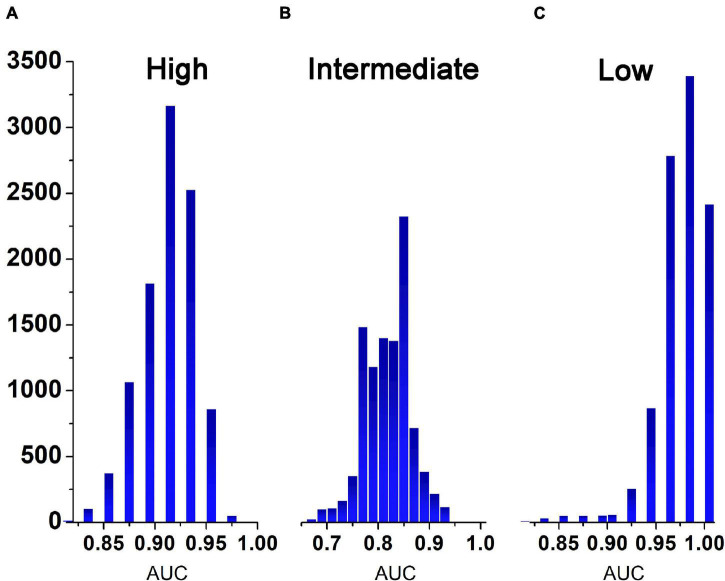
Histogram representing the frequency of AUCs obtained after 10,000 tests. **(A)**, high risk group; **(B)**, medium risk group; **(C)**, low risk group.

The representative values of the AUCs obtained after 10,000 tests using the learned ANN classifier developed in this study were 0.92 for the high-risk level, 0.83 for the intermediate-risk level, and 0.98 for the low-risk level. The median value in the histogram in Figure 2 is the representative value of AUC, and the 95% range of the confidence interval of the dataset is the verification range. As for the classification accuracy of the logistic regression model presented by Li et al. (2019), the representative AUC of the high-risk level was 0.856 and the representative AUC of the low-risk level was 0.86. Methodologically, the AUC of the intermediate level could not be predicted. Therefore, the accuracy of the ANN classifier developed in this study was 6.4% higher for the high-risk level and 12% higher for the low-risk level than the accuracy suggested in a previous study (Li et al., 2019). The minimum value of the confidence interval for the ANN classifier was 4% higher for the high-risk level and 9% higher for the low-risk level while the maximum value in the confidence interval for the ANN classifier was 10% higher at the high-risk level and 10.5% at the low risk level (Table 2).

**TABLE 2:** In this study, a comparison of the accuracy of prediction of Tdp-induced risk levels when using an artificial neural network (ANN) model and a logistic regression model proposed by the Li group was performed (Li et al., 2019).

Model	Logistic regression	ANN
AUC of High risk group	0.86 (0.81–0.9)	0.92 (0.85–0.96)
AUC of Intermediate risk group	—	0.83 (0.73–0.91)
AUC of Low risk group	0.86 (0.82–0.90)	0.98 (0.91–1)
Likelihood + of High risk group	5 (3.33–12.5)	5,000 (4,000–6,000)
Likelihood − of High risk group	0.556 (0.278–0.588)	0.5 (0.40–0.59)
Likelihood + of Intermediate risk group	—	2.249 (1.80–2.25)
Likelihood − of Intermediate risk group	—	0.18e-3 (0.18e-3–0.26)
Likelihood + of Low risk group	2.01 (1.61–2.84)	6,000 (4.39–6,000)
Likelihood − of Low risk group	0.118 (1.8e-06–0.284)	0.4 (0.4–0.66)

The positive likelihood ratio, which classifies the high-risk groups, has a median value of 5 for the positive likelihood ratio degree in the regression model and 5,000 for the ANN model. The regression model was five times more likely to classify high-risk groups as high-risk groups than the other risk groups. The ANN model is 5,000 times more likely to classify high-risk groups as high-risk groups than other risk groups. The negative likelihood ratio was 0.556 in the regression model while it was 0.5 in the ANN model. The regression model was 1.8 times less likely to classify high-risk groups into other risk groups than it did classify high-risk groups. The ANN models was twice as likely to classify high-risk groups as intermediate-risk or low-risk groups. The positive likelihood ratio in the intermediate-risk group was 2.249 and the negative likelihood ratio was 1.8 × 10^–4^ in the ANN model. This was not presented in the regression model. In the ANN models, the likelihood of classifying an intermediate-risk group as an intermediate-risk group was 2.249 times higher than that of other risk groups, and 5.6 × 10^3^ times less likely to classify the intermediate-risk group as a different risk group.

The positive likelihood ratio in the low-risk classification is 2.01 in the regression model and 6,000 in the ANN model. In regression models, the likelihood of classifying low-risk groups as low-risk groups was twice as likely as that of other risk groups. In ANN models, the likelihood of classifying low-risk groups as low-risk groups was 6,000 times more likely than that of other risk groups. The regression model has a negative likelihood ratio of 0.118 and a ANN model has negative likelihood ratio of 0.4. In the regression model, it was 8.5 times (1/LR-) less likely to classify low-risk groups into other risk groups. ANN models were 2.5 times less likely to classify low-risk groups into other risk groups (Table 2).

## Discussion

In this study, a ANN model was developed to evaluate drug cardiotoxicity by inputting nine multiple features values, including AP morphology (APD90, APD50, dVm/dt_max_, and AP_resting_), calcium transient morphology (CaD90, CaD50, and Ca_resting_), and charge features (qNet and qInward). An *in silico* simulation using the Dutta model was performed to derive nine features values. As for the performance of the classification algorithm, when comparing the results through the same *in silico* model and validation method as performed by Li et al., our performance was 10.2% higher at the high-risk level and 6.7% higher in the low-risk level compared to the classification performance presented by Li et al. In addition, by using an ANN instead of the logistic regression classification method used by Li et al., it was possible to explicitly classify not only high-and low-risk levels, but also intermediate-risk levels.

We selected 9 TdP prediction biomarkers as input indicators of the ANN model. The 9 indicators were selected from 13 indicators (dVm/dt_max_, Vm_peak_, AP_resting_, APD90, APD50, APD_triangulation_, Ca_peak_, CaD90, CaD50, CaD_triangulation_, qNet, and qInward) from previous studies. The four excluded indicators are Vm_peak_, the peak value of the AP, and Ca_peak_, the peak value of the Calcium transient shape, and APD triangulation, the difference between APD90 and APD50, and Ca triangulation, the difference between CaD90 and CaD50. Vm_peak_ and Ca_peak_ were excluded because it relied on time steps *in silico* simulation. Since APD_triangulation_ is dependent on APD90 and APD50, and Ca_triangulation_ is dependent on CaD90 and CaD50, they were excluded because of their high correlation (May et al., 2011). So, 9 indicators were finally selected, and as a result of the test by setting input indicators in various combinations (19 cases) between nine indicators, the performance of using all nine indicators was the best (Supplementary Table 5). The index that most influences the classification performance of high-risk and low-risk groups is dVm/dt_max_, and the performance of eight indicators excluding this indicator was 7% lower in the high-risk group and 27% lower in the low-risk group than in the results of using nine indicators. The index that most affects the classification performance of the intermediate-risk group was qInward, and when this indicator was excluded, the performance of the intermediate-risk group decreased by 37%. Through this analysis, nine input indicators were finally selected as input features of the artificial intelligence classifier.

When performance was evaluated on the basis of diagnostic accuracy as proposed by Li and Šimundić (Simundic, 2009; Li et al., 2019), the AUC value was in the “excellent” accuracy range of 0.9 or higher for high-risk and low-risk groups. The intermediate-risk AUC performance was more than 0.8. in the Li group evaluation criteria range is “good,” and in the evaluation criteria of Šimundić, the range is of “very good” accuracy. The accuracy of LR+ was higher than 10 for the high-risk and low-risk groups, which are classified as “excellent” in the Li group and were also good indicators in the criteria proposed by Šimundić. The LR+ in the intermediate-risk group is 2, which was the minimum acceptable performance on the basis of the Li group. The LR- was the least acceptable performance on the basis of the Li group in the high-risk and low-risk groups, while that of the intermediate risk group was considered “excellent.” The results show that the ANN model addresses the problem of low specificity, which was the problem faced by the hERG assay as evaluated using the existing ICH S7B guidelines (Colatsky et al., 2016; Lancaster and Sobie, 2016).

Disopyramide and azimilide in the high-risk group were classified as intermediate-risk groups. Loratadine and tamoxifen in the low-risk group were classified as intermediate, and our model predicted all four incorrectly classified drugs as intermediate. The results for each drug classified risk group are attached in Supplementary Table 6. The classification performance of qNet calculated through the model using hERG dynamic drug binding in the Li group predicted disopyramide of high-risk drugs as an intermediate-risk group (Li et al., 2019). Clozapine and risperidone of intermediate-risk drugs were indicated as low-risk groups, and domperidone was predicted as high-risk groups. Low-risk drugs metoprolol were classified as intermediate-risk groups. Li group has 11 accurately classified, and our model has 12 accurately classified. Disopyramide was a drug that was difficult for the Li group and our model to predict in common. One of the reasons why drugs are misclassified is that the influence of drugs implemented in the *in silico* model based on the IC50 and Hill coefficients does not fully implement complex pharmacokinetic reactions. In addition, since the label of TdP risk group was determined by clinical trials, it is difficult to say that the cell model fully represents the drug response at the organ-level. Misclassification was also observed in previous studies. For example, CaD90 measured through human stem cells was changed (prolonged; nitrendipine, nifedipine, or shortened; metoprolol) in three properly classified drugs among the five drugs, but no significant changes were observed in two misclassified drugs (loratadine and tamoxifen). As such, it has already been reported that drugs misclassified by the *in silico* stage are already inconsistent in the *in vitro* assay stage. In addition, disopyramide and azimilide in high-risk groups were classified as intermediate-risk groups in both regression models using Bnet (Han et al., 2019). Bnet, a TdP predictor calculated by IC50 and Hill coefficients, shows the highest performance among indicators using experimental parameters to date, but failed to correctly classify disopyramide and azimilide.

A limitation of this study is that the nine parameters should be provided as input values for the toxicity assessment classifier. The values obtained in this study were significantly more than the only one input value required in previous studies. To obtain nine reliable parameters, the sufficient reliability of the physiological/pharmacological *in silico* model of cardiomyocytes should be supported. A second limitation of this study is that, in previous studies, risk groups were classified based on the threshold values of physiologically/pharmacologically meaningful parameters, such as qNet, qInward, and T_qNet_. However, the ANN model proposed in this study does not provide an explicit threshold for such classification. This means that when the researcher uses this artificial neural network classifier for cardiac toxicity evaluation, it is difficult to evaluate the results classified through ANN based on clinical validity. The above two limitations will be inevitably encountered if an ANN-based machine learning method is used. Nevertheless, it would be highly meaningful in the field of new drug development research to develop an algorithm with higher toxicity assessment and classification performance than what has been proposed in previous studies.

## Data Availability Statement

The datasets presented in this study can be found in online repositories. The names of the repository/repositories and accession number(s) can be found below: https://github.com/Yedam-Y/ANN_CiPA/.

## Author Contributions

All authors contributed (to varying degrees) toward the analyses performed, developing the research concept, simulation design, developing the simulation source code, performing the simulation, and writing of the manuscript.

## Conflict of Interest

The authors declare that the research was conducted in the absence of any commercial or financial relationships that could be construed as a potential conflict of interest.

## Publisher’s Note

All claims expressed in this article are solely those of the authors and do not necessarily represent those of their affiliated organizations, or those of the publisher, the editors and the reviewers. Any product that may be evaluated in this article, or claim that may be made by its manufacturer, is not guaranteed or endorsed by the publisher.

## Footnotes

1
https://github.com/FDA/CiPA/
