# Supplementary material for: Assessment of Drug Proarrhythmicity Using Artificial Neural Networks With in silico Deterministic Model Outputs
Source: Front Physiol. 2021 Dec 10;12:761691. doi: 10.3389/fphys.2021.761691 (PMC8703011; doi:10.3389/fphys.2021.761691)
Supplement: Supplementary file 1 [file Data_Sheet_1.pdf]

Optimal model selection process

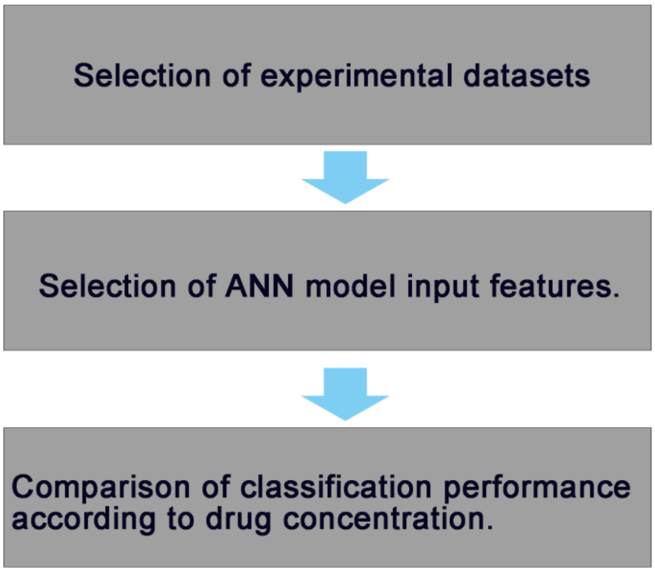

Figure S1.

We performed a process for the selection of the drug toxicity classifier model to find the best optimal model as shown in the schematic diagram. We compared the classifier's performance using two experimental data of Crumb data [1] and Chantest data [2], respectively. The classification performance was higher when using Crumb data than when using Chantest data. It was corresponded to the results of Li et al. and Zhou et al; they used the Cumb dataset to build the drug evaluation model [3], [4].

From the in silico simulation, we obatined 14 electrophysiological features; APD90, APD50, APDtrianguration (APDtri), CaD90, CaD50, CaD triangulation (CaDtri), dVm/dt_{max}, Vm peak, Vm resting, Ca transient peak, and Ca transient Resting, including qNet and qInward of the in silico model presented in Comprehensive In vitro proarrhythmia assay (CiPA). We tested the model performance by changing the combinations of input features. The combinations of input feature consisted of from 3 to 14 electrophysiological features, and the number of combinations was 13. Among the models using 13 combinations, the model using nine features of dVm/dt_{max}, APresting, APD90, APD50, Ca resting, CaD90, CaD50, qNet, and qInward has the best performance. Accordingly, we empirically selected these nine featutres as the most optimal combinations (with a minimum AUC of 0.6 or higher in all-risk groups, Table 4).

In the previous research, the Li group calculated the average value of the features at 1,2,3,4 times the drug Cmax concentration and used it as an input for the classification model. We used the average feature of Cmax 1-4 by following their protocol strictly to quantitatively compare the classification performances with them. To validate the average feature, we had tested several models using various concentration features in the development of a classification model for proarrhythmic drug risk. We compared the results of models using individual concentration features (Cmax*1 data, Cmax*2 data, and Cmax*3 data, and Cmax*4 data, respectively) and the model using summation features of Cmax 1-4 with the model using average features of Cmax 1-4. Among the six models we mentioned above, the ANN model using average features had the best performances than other models (Table 5). The data we used in this study are opened in CiPA website; Github.com/Yedam-Y/ANN.

As a result, we used the experimental data of Crumb et al. as input to the in silico model. Through the empirical research, we finally proposed an artificial neural network classification model with the averaged concentration of 9 features (dVm/dt_{max}, APresting, APD90, APD50, Ca resting, CaD90, CaD50, qNet, qInward) as input.

Table S1. Crumb et al. data IC50 and hill coefficient data information [1]

	IC50 (micro Mol)				hill coefficient			
Drug name/ ion channel	INaL	ICaL	INa	IKr	INaL	ICaL	INa	IKr
dofetilide	126 (0.015, 5.68e+06)	44.5 (0.0123 , 5.58e+06)	1.36 (0.011, 1.74e+06)	0.0014(0.0011,0.0018)	1.1 (0.15, 9.5)	3.6 (0.32, 9.6)	1.1 (0.24 , 9.1)	0.63(0.48 ,0.79)
quinidine	9.46 (7.8 , 12.4)	53.5 (29.6, 141)	12.4 (9.06, 21.5)	0.34(0.24, 0.44)	1.3 (1 , 1.7)	0.58 (0.44, 0.74)	1.5 (1 , 2.2)	1.04(0.70 ,1.47)
sotalol	3.28e+03 (2.61e+03, 6.46e+03)	7.13e+03 (4.34e+03, 1.62e+04)	1.12e+05 (3.22e+03, 7.85e+06)	91.69(59.2 , 135.9)	4.8 (1.9, 9.6)	0.87 (0.59, 1.3)	0.86 (0.39, 8.4)	0.98(0.72 ,1.54)
bepridil	1.82 (1.56, 2.12)	2.82 (1.95, 5.31)	2.96 (2.42, 4.03)	0.16 (0.097, 0.258)	1.4 (1.1, 1.8)	0.65 (0.44, 0.92)	1.2 (0.81 , 1.7)	0.96 (0.614,3.01)
cholorpromazine	4.59 (3.77, 5.57)	8.32 (6.13, 12.3)	4.58 (3.72, 6.01)	1.13(0.85, 1.55)	0.94 (0.78, 1.1)	0.85 (0.61, 1.2)	2.1 (1.6, 3.1)	0.90(0.70 , 1.23)
cisapride	9.26e+03 (3.43, 7.28e+06)	1.03e+03 (0.359, 6.36e+06)	1.79e+03 (0.277, 5.32e+06)	0.011 (0.007, 0.018)	6.3 (1.8, 9.8)	4.8 (0.41, 9.7)	0.67 (0.23, 9.4)	0.63(0.49 , 0.84)
ondansetron	19.3 (15.8, 24.7)	22.7 (16.1, 38.6)	38.5 (22.5, 301)	1.55(1.03, 2.35)	1 (0.8, 1.4)	0.76 (0.55, 1)	1.6 (0.5, 8.7)	1.00(0.71 , 1.53)
terfenadine	14.9 (1.48, 2.31e+03)	0.704 (0.612, 0.817)	1.73 (0.974, 13.9)	0.019(0.011, 0.032)	0.66 (0.27, 3.2)	0.66 (0.59, 0.74)	2.4 (0.66, 9.2)	0.60(0.44 , 0.87)
mexiletine	9.02 (7.71, 11.3)	38.9 (22.3, 114)	26.1 (13.4, 9.46e+05)	28.2(13.0, 110.9)	1.4 (1 , 1.8)	1 (0.65, 1.6)	3.8 (0.4, 9.5)	4.38 (1.43,9.68)
verapamil	24.1 (1.32, 3.44e+06)	0.204 (0.163 , 0.25)	2.59e+03 (2.51, 6.35e+06)	0.50(0.43, 0.59)	2 (0.22, 9.4)	1.1 (0.86, 1.4)	3.5 (0.37 , 9.7)	1.10(0.89 , 1.36)
ranolazine	7.94 (6.2 , 10.3)	900 (35.2, 5.88e+06)	53.3 (27.4, 538)	6.66(5.05, 9.23)	0.95 (0.72, 1.3)	3.9 (0.49, 9.6)	1.9 (0.62 , 9.1)	0.84(0.65 ,1.06)
diltiazem	21.6 (16.7, 30.8)	0.113 (0.0747, 0.167)	36.9 (15 , 385)	6.74(5.27, 8.64)	0.68 (0.55, 0.91)	0.72 (0.53, 1)	1.4 (0.47 , 8.7)	0.79(0.66 ,0.99)

Table S2. Chantest dataset IC50 and hill coefficient data information [2]

	IC50(micro Mol)				hill coefficient			
Drug name/ ion channel	INaL	ICaL	INa	IKr	INaL	ICaL	INa	IKr
dofetilide	5262000 (248100, 9683 000)	5351000 (268500, 9771 000)	4937000 (260600, 9594 000)	5198000 (241100, 9532000)	1.4 (0.57, 1 .97)	1.40 (0.55, 1. 97)	1.38 (0.55, 1. 96)	1.42 (0.57, 1.97)
quinidine	5265000 (248400 9839 000)	5452000 (266000, 9679 000)	5232000 (254000, 9908 000)	17.16(13. 5, 29.4)	1.38 (0.53, 1 .9)	1.35 (0.54, 1. 97)	1.35 (0.54, 1. 96)	1.42 (0.86, 1.95)
sotalol	4650000 (244500, 9790 000)	4771000 (254300, 9805 000)	4948000 (284400, 9735 000)	4800000 (141100, 976800)	1.40 (0.56, 1 .97)	1.41 (0.582, 1.97)	1.42 (0.59, 1. 97)	1.26 (0.53, 1.95)
bepridil	5313000(2181 00,9847000)	4842000(2351 00,9689000)	4195000(2520 00,9513000)	5328(261. 4, 9843)	1.41(0. 57,1.97)	1.36(0.5 4, 1.97)	1.32(0.5 4, 1.96)	1.42(0. 58,1.9)
clarithromycin	11.4 (3.88, 117.8)	2377000 (53040, 37080 00)	10.7 (3.26, 833.3)	3893000 (219200, 9636000)	0.98 (0.53, 1 .9)	1.256 (0.55, 1. 96)	1.19 (0.56, 1. 93)	1.31 (0.56, 1.96)
cisapride	46100000 (227700, 9843 000)	4693000 (229500, 9487 000)	4937000 (235700, 9781 000)	5891000 (241500, 9824000)	1.39 (0.55, 1 .97)	1.43 (0.58, 1. 97)	1.41 (0.56, 1. 97)	1.14 (0.53, 1.94)
ondansetron	5322000 (274700, 9789 000)	5645000 (221700, 9986 000)	4901000 (245700, 9650 000)	4603000 (218500, 8821000)	1.40 (0.59, 1 .96)	0.75 (0.50, 1. 93)	1.37 (0.55, 1. 97)	1.42 (0.583, 1.97)
terfenadine	5295000 (243600, 9841 000)	5136000 (261900, 9730 000)	4772000 (244800, 9890 000)	4784000 (259600, 9752000)	1.38 (0.578, 1.972)	1.38 (0.56, 1. 97)	1.36 (0.54, 1. 97)	1.4 (0.57, 1.96)
mexiletine	253.2 (96.13, 22670)	230 (103.9, 1311)	2645000 (5062, 588000 0)	68.5 (57.8, 85. 3)	1.24 (0.57, 1 .94)	1.0 (0.53, 1. 88)	1.35 (0.57, 1. 96)	1.26 (0.97, 1.64)
verapamil	4956000 (253000, 9476 000)	3,56 (1.98, 6.684)	4916000 (251100, 9935 000)	5314000 (226900, 9896000)	1.39 (0.56, 1 .96)	0.57 (0.50, 0. 81)	1.44 (0.59, 1. 97)	1.40 (0.57, 1.97)
ranolazine	4702000 (254100, 9729 000)	4701000 (263700, 9847 000)	4397000 (199800, 9619 000)	87.2 (60.14, 14 9.3)	1.43 (0.58, 1 .97)	1.33 (0.53, 1. 96)	1.34 (0.55, 1. 97)	1.08 (0.72, 1.6)
diltiazem	119.8 (69.1, 303)	32.4 (22.77, 45.9.)	4733000 (218300, 9786 000)	19.7 (17.6, 22. 13)	0.91 (0.53, 1 .75)	1.24 (0.77, 1. 83)	1.39 (0.55, 1. 97)	1.28 (1.12, 1.46)

Table S3. Classification performance according to experimental data.

The performance test of the ANN model was performed with two data, Chantest data[2] and Crumb data[1]. It showes high performance when in silico model as the input used crumb data. Nine features calculated using each experimental data as input of the in silico model were used as input of the ANN model. The features were an average of 1, 2, 3, and 4 times the Cmax concentration, and we used 2,000 samples according to each drug. The test result is an AUC result calculated using 10,000 datasets. Among the 10,000 test results, it was expressed as an intermediate value (CI95%).

	high	intermediate	low
Crumb data [1]	0.92(0.85-0.96)	0.83(0.73-0.91)	0.98(0.91-1)
Chantest data [2]	0.90(0.83-0.95)	0.68(0.58-0.72)	0.89(0.85-0.94)

Table S4. Performance of classifier by concentration (based on AUC)

We tested which drug concentration the nine features calculated have the highest performance. The data in the table expressed the 'median value (minimum value-maximum value)' among the tests of 10,000 datasets. Using the averaged value of Cmax 1-4 times had the highest performance.

	High	intermediate	low	Sample number /drug
Cmax* 1	0.98(0.60-1)	0.71(0.42-0.92)	0.84(0.70-0.96)	2,000/1 drug
Cmax *2	0.92(0.56-1)	0.68(0.38-0.90)	0.87(0.70-1)	2,000/1 drug
Cmax *3	0.96(0.77-1)	0.75(0.508-0.93)	0.91(0.74-1)	2,000/1 drug
Cmax *4	0.83(0.64-0.95)	0.71(0.46-0.92)	0.93(0.69-1)	2,000/1 drug
Total of Cmax 1-4	0.94(0.67-1)	0.73(0.48-0.89)	0.84(0.6-1)	8,000/1 drug
Averaged of Cmax 1-4	0.92(0.80-1)	0.83(0.69-0.95)	0.98(0.91-1)	2,000/1 drug

Table S5. Comparison of model performance according to features combination (based on AUC)

Among the nine features selected by empirical trials, we confirmed the results of using various feature combinations. Among the various combinations, we obtained the results of nine combinations except for those with a value of 0.6 or less among AUCs in high-risk, intermediate-risk, and low-risk groups. This test is a table comparing the median values through 1,000 tests.

Features combination	High	intermediate	low
APD90,CaD90,qnet	0.88	0.746	0.845
APDtri,CaD90,qnet	0.85	0.67	0.79
APD90,CaDtri, qnet	0.81	0.60	0.80
dVm/dtmax,Ca peak,qnet, qinward	0.86	0.667	0.75
APD90,CaD90,qnet,qinward	0.86	0.73	0.74
APD90,APD50,CaD90,CaD50,qnet	0.81	0.78	0.83
dVm/dtmax, APresting, APD90,CaD90, qnet	0.83	0.69	0.81
Ca resting,CaD50,APD90,CaD90, qnet	0.875	0.619	0.836
APD50,ca resting, APD90,CaD90, qnet, qinward	0.78	0.61	0.68
dV_m/dt_{max}, $AP_{resting}$, APD90, APD50, $Ca_{resting}$, CaD90, CaD50, qNet, and qInward	0.92	0.83	0.98

Table S6. The effect of each of the nine features on the performance of the model

This table is a test result table for the ANN model, which inputs eight features, excluding one out of 9 features. We compared the median values of 1,000 tests. We confirmed the effect of one of the nine features selected as optimal on the classification of high-risk, intermediate-risk, and low-risk groups.

Exception feature	high	inter	low
**APD50**	0.92	0.58	0.88
**APD90**	0.92	0.59	0.86
**Apresting**	0.88	0.65	0.83
**CaD50**	0.85	0.59	0.8
**CaD90**	0.9	0.63	0.84
**Caresting**	0.91	0.75	0.9
**dVm/dtmax**	0.84	0.68	0.71
**qinward**	0.91	0.46	0.92
**qnet**	0.9	0.51	0.872

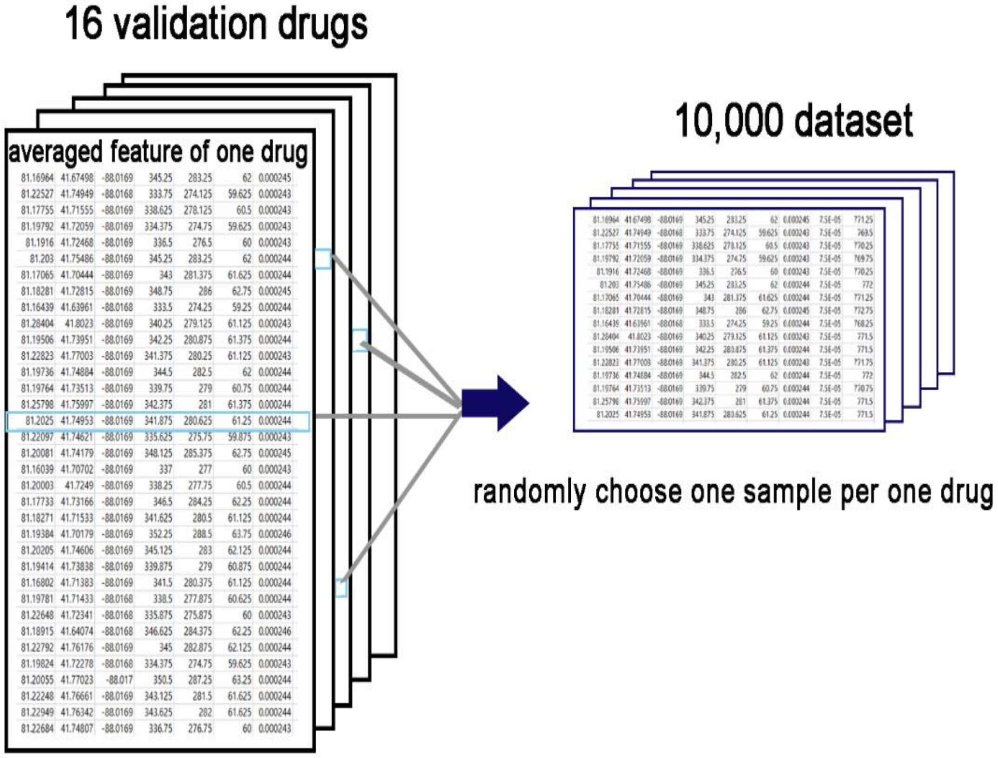

Figure S2. A single dataset is created by taking one random sample from 16 drug data (2,000/1 drug) averaged according to concentration. An example of an average of 2,000 samples with Cmax 1-4 on the left, and 10,000 samples on the right, containing 16 drug data, one made by taking one sample out of 2,000 drugs.

Table S7. Comparison table between the drug risk group and the actual risk group predicted through the test. The number of labels predicted by each drug through the model, and the probability predicted, were expressed as median (minimum to maximum) in 10,000 tests. Gray marks are misclassified drugs.

Drug name	True label	Predicted True label Probability	Number of Predicted low	Number of Predicted intermediate	Number of Predicted high
ibutilide	high	0.776(0.765-0.784)	0	0	10000
vandetanib	high	0.754(0.748-0.763)	0	0	10000
disopyramide	high	0.223(0.179-0.298)	0	10000	0
azimilide	high	0.290(0.225-0.363)	0	9991	9
risperidone	inter	0.442(0.435-0.461)	0	10000	0
domperidone	inter	0.442(0.428-0.458)	36	9964	0
droperidol	inter	0.419(0.400-0.430)	0	9883	117
clarithromycin	inter	0.438(0.427-0.450)	8	9992	0
clozapine	inter	0.425(0.411-0.428)	0	10000	0
astemizole	inter	0.434(0.422-0.440)	0	10000	0
pimozide	inter	0.440(0.431-0.490)	339	9661	0
metoprolol	low	0.877(0.871-0.883)	9579	421	0
nifedipine	low	0.619(0.609-0.631)	10000	0	0
nitrendipine	low	0.511(0.501-0.530)	9958	42	0
loratadine	low	0.415(0.403-0.421)	0	10000	0
tamoxifen	low	0.394(0.372-0.416)	0	10000	0

Reference

[1] W. J. Crumb, J. Vicente, L. Johannesen, and D. G. Strauss, “An evaluation of 30 clinical drugs against the comprehensive in vitro proarrhythmia assay (CiPA) proposed ion channel panel,” *J. Pharmacol. Toxicol. Methods*, vol. 81, pp. 251–262, 2016, doi: 10.1016/j.vascn.2016.03.009.

[2] X. Han *et al.*, “A general procedure to select calibration drugs for lab-specific validation and calibration of proarrhythmia risk prediction models: An illustrative example using the CiPA model,” *J. Pharmacol. Toxicol. Methods*, vol. 105, no. June, p. 106890, 2020, doi: 10.1016/j.vascn.2020.106890.

[3] Z. Li *et al.*, “Assessment of an In Silico Mechanistic Model for Proarrhythmia Risk Prediction Under the CiPA Initiative,” *Clin. Pharmacol. Ther.*, vol. 105, no. 2, pp. 466–475, 2019, doi: 10.1002/cpt.1184.

[4] X. Zhou *et al.*, “Blinded in silico drug trial reveals the minimum set of ion channels for torsades de pointes risk assessment,” *Front. Pharmacol.*, vol. 10, no. January, pp. 1–17, 2020, doi: 10.3389/fphar.2019.01643.
